# Evaluation of the Irritable Bowel Syndrome Quality of Life (IBS-QOL) questionnaire in diarrheal-predominant irritable bowel syndrome patients

**DOI:** 10.1186/1477-7525-11-208

**Published:** 2013-12-13

**Authors:** David A Andrae, Donald L Patrick, Douglas A Drossman, Paul S Covington

**Affiliations:** 1 Furiex Pharmaceuticals, Inc., 3900 Paramount Parkway, Suite 150, Morrisville, NC 27560, USA; 2 Department of Health Services, University of Washington School of Public Health, Seattle, WA, USA; 3 Drossman Center for the Education and Practice of Biopsychosocial Care LLC, and UNC Center for Functional GI and Motility Disorders, Chapel Hill, NC, USA

**Keywords:** IBS-QOL, Patient-reported Outcomes, Psychometrics, HRQOL, Irritable Bowel Syndrome, Diarrhea, Eluxadoline

## Abstract

**Background:**

Diarrhea-predominant irritable bowel syndrome (IBS-d) significantly diminishes the health-related quality of life (HRQOL) of patients. Psychological and social impacts are common with many IBS-d patients reporting comorbid depression, anxiety, decreased intimacy, and lost working days. The Irritable Bowel Syndrome Quality of Life (IBS-QOL) questionnaire is a 34-item instrument developed and validated for measurement of HRQOL in non-subtyped IBS patients. The current paper assesses this previously-validated instrument employing data collected from 754 patients who participated in a randomized clinical trial of a novel treatment, eluxadoline, for IBS-d.

**Methods:**

Psychometric methods common to HRQOL research were employed to evaluate the IBS-QOL. Many of the historical analyses of the IBS-QOL validations were used. Other techniques that extended the original methods were applied where more appropriate for the current dataset. In IBS-d patients, we analyzed the items and substructure of the IBS-QOL via item reduction, factor structure, internal consistency, reproducibility, construct validity, and ability to detect change.

**Results:**

This study supports the IBS-QOL as a psychometrically valid measure. Factor analyses suggested that IBS-specific QOL as measured by the IBS-QOL is a unidimensional construct. Construct validity was further buttressed by significant correlations between IBS-QOL total scores and related measures of IBS-d severity including the historically-relevant Irritable Bowel Syndrome Adequate Relief (IBS-AR) item and the FDA’s Clinical Responder definition. The IBS-QOL also showed a significant ability to detect change as evidenced by analysis of treatment effects. A minority of the items, unrelated to the IBS-d, performed less well by the standards set by the original authors.

**Conclusions:**

We established that the IBS-QOL total score is a psychometrically valid measure of HRQOL in IBS-d patients enrolled in this study. Our analyses suggest that the IBS-QOL items demonstrate very good construct validity and ability to detect changes due to treatment effects. Furthermore, our analyses suggest that the IBS-QOL items measure a univariate construct and we believe further modeling of the IBS-QOL from an item response theory (IRT) approach under both non-treatment and treatment conditions would greatly further our understanding as item-based methods could be used to develop a short form.

## Background

Irritable bowel syndrome (IBS) affects an estimated 10-15% of people in western cultures [1]. It is characterized by recurrent abdominal pain and diarrhea [2] and can negatively impact health-related quality of life (HRQOL). IBS subtypes are defined by Rome III and include: diarrhea (IBS-d), constipation (IBS-c), or mixed constipation and diarrhea (IBS-m) [3]. Safe and effective pharmacologic treatments for IBS are limited, with current treatment options including antispasmodics, antidepressants, antidiarrheal agents, and alosetron [4]. Recently, clinical outcome results from a large Phase 2 clinical trial in IBS-d patients of a novel mixed mu-opioid (μ-OR) agonist, delta-opioid (δ-OR) antagonist, eluxadoline, were reported [5]. Additionally, HRQOL instruments were included in the study as secondary outcomes. By subjecting this larger data set to procedures outlined by previous publications [6,7] we plan to confirm the original psychometric validation analyses of the non-subtyped IBS-QOL. More specifically, we hope to assess how the IBS-QOL performs in strictly IBS-d patients.

### Historical development and validation of the IBS-QOL

Patrick, et al., describe the steps utilized for item construction to ensure content validity of the IBS-QOL items [6,8] in which a combination of forty IBS-d, IBS-c, and IBS-m patients were interviewed resulting in identification of 117 potential items to describe these patients’ IBS. Next, 30 additional patients underwent cognitive debriefing interviews which led to the retention of 45 items from the pool. After review by HRQOL and gastroenterology experts from Europe 41 items were found to be sufficiently content valid for use in the United States, Britain, Germany, Italy, and France. This pilot questionnaire was then mailed to 169 patients who: a) met Rome criteria for IBS, [9] b) were symptomatic at least 2 days/week, and c) were aged 18 to 65; 156 patients responded: 60% IBS-m, 22%, IBS-c, and 19% IBS-d.

Drossman, et al., extended the validation of the IBS-QOL by collecting questionnaires from 156 females with a functional bowel disorder [7]. These patients had moderate to severe symptoms ≥ 2 days/week over 3 months. Patients were assessed the two weeks prior to start of treatment and again after 12 weeks. For abdominal pain, visual analog reports were averaged over the two-week intervals from Week-2 to treatment start and Weeks 11-12.

Drossman, et al., [10] further investigated the IBS-QOL’s ability to detect treatment changes. After utilizing pain and treatment satisfaction as anchors for interpreting IBS-QOL total scores from a sample of mostly female IBS patients, the authors concluded that a 14-point improvement in IBS-QOL scoring was clinically meaningful. In the current paper, we will examine the IBS-QOL total score improvement keeping the Drossman, et al., improvement of 14-points as a historical reference point.

Psychometric evaluations utilized in these studies included:

• Item reduction.

• Factor structure analysis employing principal components analysis (PCA) with orthogonal rotation using the varimax method.

• Internal consistency reliability assessed by Cronbach’s Coefficient α.

• Reproducibility via comparing the overall IBS-QOL score at Baseline and one week later using the Intraclass Correlation Coefficient (ICC).

• Construct validity determined by correlational analyses for convergence and divergence of IBS-QOL scores with other clinical measures.

• Ability to detect change assessed by statistical comparison of change scores in the IBS-QOL in response to treatment.

• Comparisons among a priori determined responder groups to aid in the interpretation of the IBS-QOL changes in scores due to treatment effects.

Additional file 1 contains the final instrument of 34 items consisting of 8 subscale domains determined by the original work. Previous research has demonstrated that the IBS-QOL is internally consistent, highly reliable, has convergent and divergent validity, and acceptable responsiveness to treatment effects.

## Methods

The goal of the current paper is to replicate the original IBS-QOL validation by using similar methodologies as previous efforts but with attention specifically to the IBS-d patient population [6,7]. Psychometric and statistical techniques were applied to 753 patients aged 18-65 years who completed the IBS-QOL at their Baseline visit. Between May 2010 and April 2011, 292 study centers obtained informed consent enrolled 807 patients into the study, with women representing approximately 70% of patients. A list of study investigators appears in Additional file 2. All patients all met the Rome III criteria for IBS-d [2], were compliant with their daily diary during the week prior to randomization, received at least one dose of double-blind study medication, and had at least one post-randomization diary entry. They also had to meet minimal requirements for presence of abdominal pain and stool consistency ratings. Patients were required to complete the electronic diary on 6 of the 7 required days during the week prior to randomization AND on 11 of the 14 required days during the 2 weeks prior to randomization. Patients who were compliant in completing the screening diary on a daily basis on 6 of the 7 required days during the week prior to randomization AND on 11 of the 14 required days during the 2 weeks prior to randomization, had an average of daily worst abdominal pain ratings of 3.0 or greater over the previous week, had a weekly mean Bristol Stool Score of 5.5 or greater over the previous week, and who had not used any rescue medication in the preceding 2 weeks were eligible for participation and immediate randomization into the double blind treatment phase. Conduct of the trial was overseen by Institutional Review Boards and complied with the Declaration of Helsinki. Patients in the trial completed the IBS-QOL, as well as other outcome measures, over 3 months while receiving either placebo or an active dose of eluxadoline (5, 25, 100, or 200 mg) twice daily [5]. HRQOL instruments included the IBS-QOL, Adequate Relief (IBS-AR), and IBS-Symptom Severity Score (IBS-SSS) [11]. These were collected periodically and Dove, et al., detail further the design and conduct of the trial. For the trial data, many of the psychometric assessments were applied to baseline data so that possible treatment effects did not confound the results. Other assessments were made in the presence of treatment effects and these are described below.

## Assessments of pretreatment measurements

The potential for item reduction was assessed by applying the item-inclusion criteria from the original validation study [6]. Criteria were applied to the IBS-QOL instrument to see if differences exist between the current large IBS-d sample and the smaller, original, non-subtyped IBS validation sample. The criteria assessed included:

• 50% of patients responded “not at all” and therefore could not improve on the item

• 5% or more missing data

• an item-to-total correlation of <0.4 indicating that the item may be measuring a different latent construct

• pairwise correlations between individual items that exceeded 0.7 indicating redundancies in measurement.

The original factor structure of the IBS-QOL and possible alternative subscale structures were assessed. Several Confirmatory Factor Analysis (CFA) models were fit via maximum likelihood. Diagrams outlining the different conceptual models are included in Additional file 3: Figure S1. The first model corresponds to the original PCA and assumes orthogonal factors adequately measure independent subdomains of IBS-related QOL. The likelihood ratio χ^2^, Akaike Information Criterion (AIC), and Schwartz’s Bayesian Information Criterion (BIC) were used as indicators of model fit; smaller values are generally considered better. A second, hierarchical CFA was fit which imposed the original structure, but also assumes that the subscales themselves form a generalized factor, [12] presumably, the latent construct of HRQOL in IBS. Hierarchical factor analyses employ a two-step approach; the items are grouped into factors and then the factors are submitted to factor analysis. The third and fourth models fit were confirmatory bi-factor models [13]. The bi-factor approach was employed to refine the strictly hierarchical HRQOL conceptualizations as this method allows for a structure whereby subscales may explain variance not necessarily associated with the general QOL factor. Items were organized into 8 subscales and a general factor with each item having hypothesized relationships to one subscale and the general factor [14]. For example, Item 12 is hypothesized to load onto the Sexual subscale and also the general construct of HRQOL in IBS. The two bi-factor models included: one with orthogonal factors, i.e., in which the model does not allow factors to correlate with one another; and an oblique one in which correlations between factors are allowed. A single factor model in which all items load onto a single factor was also fit for reference to the other CFA models. Such a model imposes a structure in which all items load onto one general factor representing HRQOL in IBS.

Because CFA models involve fitting complex multivariate data, model fit is evaluated by inspecting several fit indices [15]. Numerous fit indices have been suggested, but minimally, a CFA model should be evaluated for fit based on a combination of different indices [16] as each assess different aspects of the model. Indices fit included:

• Goodness of Fit Index (GFI). An analogue to the R2 in regression where higher values are considered better. GFI values of 1.0 indicate perfect fit in that all observed variance is accounted for by the proposed model [17].

• Comparative Fit Index (CFI). This index assesses the amount of variance the model fits above and beyond a null model, i.e., one with no structure [17]. The CFI has a range of 0 to 1 and generally should be above 0.9 for good fit.

• Root Mean Square Error of Approximation (RMSEA). This index assesses the fit of the model according to a noncentral χ^2^ distribution that is determined by the degrees of freedom of the model [17]. Values range from 0 to infinity with smaller values being better; less than 0.05 indicates excellent fit, greater than 0.1 indicates poor fit.

• Assessment of the model residuals was also done; good fit is indicated by values between-0.1 and 0.1.

To investigate possible misspecification of any CFA models, an Exploratory Factor Analysis (EFA) of the items was conducted to evaluate what structure would be suggested by the current data sample. EFA imposes no a priori structure to the data [12] and is similar to the approach taken by Patrick, et al. [6].

So-called internal consistency reliability was assessed by computing Coefficient α for the 34-item IBS-QOL total score as well as the Coefficient α-value for all (n-1) combinations, i.e., the so-called α-if-item-deleted, to gauge influence of single items. Values of α above 0.7 indicate a good level of consistency with values above 0.9 being considered excellent. Extremely high values can call into question whether scale items could be eliminated because of redundancy.

### Assessments including postbaseline measurements

Consistency of the IBS-QOL total score over time is usually evaluated by correlating responses over repeated measurements. All administrations of the IBS-QOL post Baseline were in the presence of treatment, thus, a traditional ICC would be biased by treatment in the current case. To account for treatment effects and time trajectories on the IBS-QOL total score, reliability was assessed by first estimating variances via a linear model and utilizing the resultant conditional variances to establish reliability. Such an approach has been developed and described in two papers by Laenen, et al. [18,19]. Their reliability measures, R_Λ_ and R_T_, utilize estimated variances from a linear model to calculate reliability over the set of repeated measurements, conditional on the covariates. Thus, in lieu of calculating an ICC, reproducibility was assessed via fitting longitudinal models to the repeated administrations of the IBS-QOL accounting for treatment effect over the treatment period. Details of the approaches are given in Additional file 4.

Construct validity of the IBS-QOL total score was assessed by evaluating it in relation to other clinical outcomes. For the IBS-SSS and EQ-5D, Pearson correlations at Baseline and Week 12 were calculated. Since the scale of the IBS-SSS is opposite to that of the IBS-QOL, a negative correlation between it and the IBS-QOL total score indicates convergence. A positive correlation with the EQ-5D indicates IBS-QOL converging with general HRQOL.

Further, change from Baseline to Week 12 in IBS-QOL total scores were correlated with similar changes from Baseline for IBS-SSS, EQ-5D, and average worst abdominal pain (WAP) [7]. The change score for the WAP variable was calculated as the average of WAP ratings for Weeks 11and12 compared to the average for the two weeks prior to dosing and the average of WAP for Week 12 compared to the average in the week prior to dosing.

Additionally, correlations between the IBS-QOL total score with the IBS-AR and FDA Clinical Responder status were calculated. The IBS-AR is a historically-used global measure of change used for assessing relief in IBS. A single item, “Over the past week have you had adequate relief of your IBS symptoms?” is administered to the patient and they respond either “Yes” or “No.” Despite its established value as an endpoint measure for clinical trials dissatisfaction by regulatory agencies with the IBS-AR has led to the desire to develop quantifiable symptom based patient-reported outcome (PRO) measures for IBS [20]. Pending the development of a final IBS PRO, the FDA issued a guidance document in 2012 for drug development in IBS in which they formulated responder analysis definitions based on diary collection of pain and stool consistency ratings. One of the FDA Clinical Responder definitions from the Guidance, utilized by Dove, et al., is also used in the current paper as an additional criterion for assessing the validity of the IBS-QOL total score [5]. The definition is based on a percentage of days a patient has a simultaneous improvement in both pain and stool consistency on the same day—the so-called daily responder definition [21]. Since these outcomes are measured on a dichotomous scale two different biserial correlation approaches were calculated to account for non-continuous variables [22,23]. See Additional file 5 for a full description of the approaches.

The IBS-QOL was previously assessed for responsiveness [7] using Cohen’s d statistic [24]. In that analysis, effect sizes for the change in scores from pre-treatment to post-treatment were computed similarly to standardize mean differences by putting changes in scores into standard deviation units. The d statistic originally employed a standard deviation value that was either based on the Baseline pooled data or on the control group only. Both of these methods inherently assume homogeneity of variance, either across time points or across treatment groups. To account for potential heterogeneity of variance across treatment groups and also handle data dependencies due to repeated patient measurements an additional assessment was calculated by estimating a longitudinal model for the change in IBS-QOL total score between Baseline and Week 12 administrations. To visually assess the IBS-QOL total score, the cumulative proportion of patients meeting a certain change from Baseline to Week 12 was also plotted by treatment group and the proportion of patients meeting certain thresholds of improvement for Placebo and Eluxadoline 100 mg treatment groups were compared.

Statistical analyses were performed with R version 3.0, [25] R-package ltm, [26] and SAS® software version 9.3 [27].

**Trial registration:** ClinicalTrials.gov identifier NCT01130272

## Results

Of the 754 patients included for analysis, 526 were female and 646 were Caucasian. The mean (standard deviation) age was 44.8 (11.93) with Baseline IBS-QOL total scores averaging 53.2 (21.09).

### Assessments of pretreatment measurements

For Baseline data, Table 1 displays items that warrant further investigation as they did not meet the original qualitative criteria for inclusion. Of note, while several items displayed relatively high inter-item correlations, two items, 32 and 33, had a majority of patients respond with the, “not at all” category. Item 29, notably, had the opposite problem where many patients responded with the highest category, “a great deal.” While not restrictive in the sense that patients cannot improve on this item, such a skewed distribution could cause problems with other items when scoring or modeling is conducted. Such results are also indicative that either a reduced response set is adequate. For example, in our dataset 46.4% patients reported “not at all” for Item 33 (“My bowel problems are affecting my closest relationships”), so simply providing a “yes” versus “no” response set to this item may be adequate for IBS-d patients. Alternatively, the items may not be helpful in measuring the latent construct of interest in IBS-d patients.

**Table 1:** **Potentially problematic items according to criteria in Patrick, et al.**[6]

**Item**	**Reason(s) why item is problematic**
6. I feel like I’m losing control of my life because of my bowel problems.	• High item-to-item correlation: r_6,7_ = 0.732
	• High item-to-item correlation: r_6,10_ = 0.702
7. I feel my life is less enjoyable because of my bowel problems.	• High item-to-item correlation: r_7,6_ = 0.732
9. I feel depressed about my bowel problems.	• High item-to-item correlation: r_9,10_ = 0.707
10. I feel isolated form others because of my bowel problems.	• High item-to-item correlation: r_10,6_ = 0.732
	• High item-to-item correlation: r_10,9_ = 0.707
12. Because of my bowel problems, sexual activity is difficult for me.	• High item-to-item correlation: r_12,20_ = 0.741
20. My bowel problems reduce my sexual desire.	• High item-to-item correlation: r_20,12_ = 0.741
29. It is important to be near a toilet because of my bowel problems.	• High item-to-item correlation: r_29,30_ = 0.708
	• Potential ceiling effect: 38.9% of patients reporting, “a great deal”
30. My life revolves around my bowel problems.	• High item-to-item correlation: r_30,29_ = 0.708
32. I fear I won’t be able to have a bowel movement.	• Floor effect: 72.8% of patients reporting “not at all”
	• Low item-total correlation: r_32,Total_ = 0.292
33. My bowel problems are affecting my closest relationships	• Potential floor effect: 46.4% of patients reporting “not at all”

Table 2 displays the various fit statistics for the CFA models. Moderately good CFA fits were observed with the exception of the single factor model. In general, models should demonstrate GFI values similar to R^2^ in regression and trending above 0.85 or so and CFI—an index that compares the fitted model to a null base model—should be above 0.9. For both these indices, larger values are better. Also, findings from the RMSEA, where smaller values are better, suggest moderately-good fits since results fall between 0.05 and 0.1. Additionally, Table 2 presents both the average residual size and the percentage of residual values that fall outside of the (-0.1, 0.1) interval.

**Table 2:** Comparison of confirmatory factor model fit statistics

**Statistic**	**Subscales only**	**Hierarchical**	**Orthogonal Bi-factor**	**Oblique Bi-factor**	**Single factor**
χ^2^	2441.1	2616.9	2254.9	1923.3	3755.9
(df)	(499)	(519)	(496)	(468)	(527)
AIC	2633.1	2768.9	2452.9	2193.3	3891.9
BIC	3075.7	3119.4	2909.3	2815.7	4205.5
GFI	0.8232	0.8088	0.8346	0.8616	0.7378
CFI	0.877	0.8671	0.8889	0.9073	0.7955
RMSEA	0.072	0.074	0.069	0.066	0.091
(95% CI)	(0.070, 0.075)	(0.071, 0.077)	(0.066, 0.072)	(0.063, 0.069)	(0.089, 0.094)
Average Residual	0.0588	0.062	0.053	0.0448	0.0632
% of Residuals ≥ 0.1 or ≤ -0.1	18.7%	21.2%	16.0%	10.5%	20.0%

Note: χ^2^ = Likelihood ratio χ^2^; df = χ^2^ degrees of freedom; AIC = Akaike’s Information Criterion (smaller is better); BIC = Schwartz’s Bayesian Information Criterion (smaller is better); GFI = Goodness-of-Fit Index (Acceptable fit > 0.9); CFI = Bentler’s Comparative Fit Index (Good fit > 0.9); RMSEA = root-mean-square error of approximation (Very good fit <0.05;Moderate fit between 0.05 and 0.1); CI = confidence interval.

As with the CFA models, the EFA model was fitted for Baseline values of the IBS-QOL. The goal of the EFA was to elucidate any moderate fit of the CFAs to the data. The factor pattern loadings for the EFA solutions are compared with the subscales in Table 3; three of eight original subscales correspond one-to-one with the substructures of the data, i.e., “Body Image”, “Food Avoidance”, and “Sexual”; the other five factors only approximately fit the original substructure. The most notable departure is with Factor 1, which is a mixture of items from the “Social Reaction”, “Relationship”, and sporadic items from other subscales.

**Table 3:** Comparison between original subscale structure and data-suggested structure

	**EFA Model with Varimax Rotation (Eigenvalues)**					
**Original subscale**	**Factor 1**	**Factor 2**	**Factor 3**	**Factor 4**	**Factor 5**	**Factor 6**
	**(37.8)**	**(3.20)**	**(2.48)**	**(1.76)**	**(1.46)**	**(1.24)**
Dysphoria	13, 16	1, 6, 7, 9, 10	30			
Interference with activity		18	3, 27, 29, 31	22	19	
Body image				5, 21, 25, 26		
Health worry	32			4	15	
Food avoidance					11, 23, 28	
Social reaction	14, 17, 34			2		
Sexual						12, 20
Relationship	8, 24, 33					

Note: Eigenvalues for the extracted factors are in parentheses and higher values represent higher amounts of the variance observed in the data captured by a given factor.

Coefficient α analysis of the IBS-QOL total score demonstrated a high level of reliability between items of the IBS-QOL [α = 0.963, 95% confidence interval (0.959, 0.966)]. Additional file 6: Table S1 lists the full α item analysis. All but Item 32 (“I fear I won’t be able to have a bowel Movement”) had item-to-total correlations above 0.5. The α-value with Item 32 included is still extraordinarily high without deleting this item, however. In fact, deleting any one item does not reduce the Coefficient α-value by more than 0.003. Further, the average item-to-total correlation was r = 0.642, indicating a high consistency between item responses.

### Assessments including postbaseline measurements

The longitudinal model showed that both R_Λ_ and R_T_ had very good reliability for the data with R_Λ_ = 0.89 and R_T_ = 0.76. By comparison, Patrick, et al., reported an ICC between administrations of 0.86. This indicates the stability of IBS-QOL total scores when treatment and time effects are taken into account as well as consistency with previous assessment.

Correlations between IBS-QOL total score and change scores for IBS-SSS, EQ-5D, 14-day WAP, the 7-day WAP, IBS-AR, and the FDA Clinical Responder values are presented in Table 4. All correlations show statistical significance. Several show moderate to strong linear relationships (r ≥ 0.40) between other variables and the IBS-QOL total score. Further, all correlations were in the correct direction given the scales of measurement of the variables.

**Table 4:** Partial correlations between IBS-QOL total score and other clinical measures—controlling for dose level

**Variable**	**Time point**	**N**	**Correlation type**	**Correlation value**	**p-Value**
IBS-SSS	Week 12	427	Pearson	-0.604	<0.0001
	Change from Baseline	383	Pearson	-0.629	<0.0001
EQ-5D	Week 12	694	Pearson	0.581	<0.0001
	Change from Baseline	690	Pearson	0.404	<0.0001
Pain/14-Day	Week 12	550	Pearson	-0.392	<0.0001
	Change from Baseline	549	Pearson	-0.350	<0.0001
Pain/7-Day	Week 12	536	Pearson	-0.397	<0.0001
	Change from Baseline	535	Pearson	-0.357	<0.0001
IBS-AR	Week 12	693	Point Biserial	0.379	<0.0001
			(t-Test)	(10.788)	
			Biserial	0.483	<0.0001
			(t-Test)	(14.523)	
FDA clinical responder definition	Week 12	694	Point Biserial	0.282	<0.0001
			(t-Test)	(7.750)	
			Biserial	0.395	<0.0001
			(t-Test)	(11.316)	

Note: Point biserial and biserial correlation coefficients are evaluated via constructing a t-test.

Table 5 displays the analyses of treatment effects detected by the IBS-QOL. Similar patterns of discrimination are observed whether calculations were conducted on the observed changes from baseline or estimated via longitudinal modeling. Additionally, effect sizes based on the pooled standard deviation between groups evaluated for the standard deviation of the Placebo group at Baseline revealed the same pattern with effect size estimates being of similar magnitude. The attenuation of effect sizes relative to Placebo was expected as the standard deviation of the Placebo group at Baseline is expected to be larger. Further, the larger disparity observed in the longitudinal model between pooled- and placebo-based effect size estimates was also expected as the estimate for the pooled standard deviation in the longitudinal model takes repeated measurement information into account.

**Table 5:** Analysis of IBS-QOL Responsiveness

	**Treatment group**	**n**	**Mean change from baseline**	**Mean difference from placebo**	**t-Value**	**Degrees of freedom**	**p-Value**	**Effect sizes**	
								**d**_**Pooled**_	**d**_**Placebo**_
IBS-QOL total score, Observed Data(a)	5 mg	66	19.73	2.02	0.64	189	0.522	0.09	0.09
	25 mg	135	18.75	1.04	0.43	258	0.664	0.05	0.05
	100 mg	130	25.25	7.54	3.10	253	0.002	0.39	0.33
	200 mg	107	25.06	7.35	2.68	230	0.008	0.35	0.32
	Placebo	125	17.70						
IBS-QOL total score, Longitudinal Model(b)	5 mg	105	19.33	2.00	1.09	262	0.279	0.13	0.09
	25 mg	167	18.46	1.13	0.67	324	0.507	0.07	0.05
	100 mg	163	24.53	7.20	4.20	320	<.001	0.47	0.31
	200 mg	160	23.33	6.00	3.64	317	<.001	0.41	0.26
	Placebo	159	17.33						

Note: (a) Effect size estimates based on observed standard deviations (s_Pooled_, s_Placebo_) (b) Effect size estimates based on estimated standard deviations from a mixed-effects model for IBS-QOL total score with fixed effects of treatment, time, treatment by time interaction, and Baseline IBS-QOL total score and random effects for intercept and time $\left({\hat{\sigma }}_{\mathrm{Pooled}},{\hat{\sigma }}_{\mathrm{Placebo}}\right)$.

**Figure 1:**
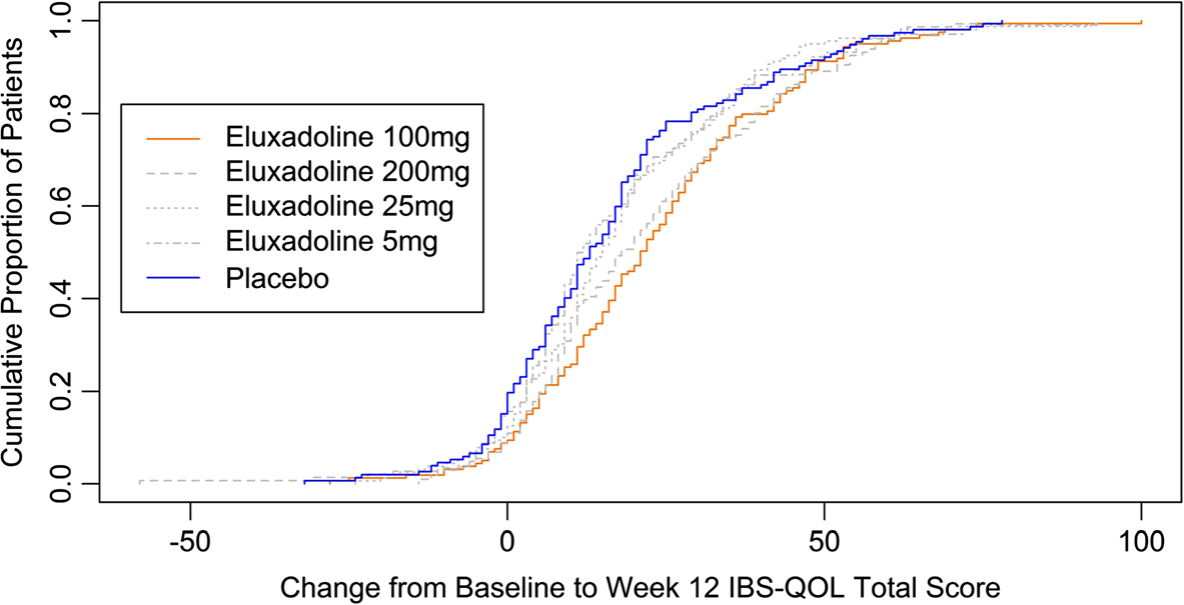
Cumulative proportions of patients meeting a certain change from Baseline to Week 12 in IBS-QOL total scores plotted by treatment group.

Figure 1 displays the cumulative proportion of patients meeting a certain change from Baseline by treatment group. Consistent with previous results [5] the higher dose groups demonstrated better improvements in IBS-QOL total score. Table 6 further elucidates the discrimination between Placebo and Eluxadoline 100 mg treatment groups. For this pairwise comparison, higher proportions of Eluxadoline-treated patients were observed for a wide range of improvement levels. Over 80% of placebo patients and over 90% of patients treated with Eluxadoline 100 mg had the same score or higher at Week 12 as compared to Baseline. Furthermore, this approximate 10% difference between these two treatment groups persisted or increased if the criterion was raised all the way up to 30 points of improvement. Interestingly, the 14-point clinically meaningful difference [10] was met by 48% of the placebo patients and over 65% of eluxadoline 100 mg patients and the maximum group difference was observed for a 22 point improvement in which over 25% of placebo patients and over 47% of eluxadoline 100 mg patients responded, respectively, for a group difference of 21.5%.

**Table 6:** Percentages of patients meeting different levels of IBS-QOL total score improvement

**Improvement from baseline to Week 12**	**Placebo**	**Eluxadoline 100 mg**	**Difference between treatment groups**
0	80.3%	90.6%	10.30%
5	70.4%	80.5%	10.11%
10	57.9%	74.2%	16.32%
15	46.1%	62.9%	16.84%
20	32.2%	52.8%	20.59%
25	21.7%	41.5%	19.80%
30	19.1%	30.8%	11.74%

## Discussion

The goal of the current paper was to replicate and expand on the original psychometric assessment of the IBS-QOL when applied to an IBS-d-specific patient set. Our results indicate that male and female IBS-d patients who are highly compliant with daily diary entry and who have a minimal requirement for pain as well as explicit criteria for diarrhea as defined by the BSS share commonalities with a general population of non-subtyped IBS patients, but that the originally-proposed subscale structure doesn’t apply as well as one might anticipate to our patient set. The deviations observed from the original assessment could be attributed to the fact that we evaluated IBS-d patients or due to the much larger sample size employed here. Without such large-scale data on other IBS subtypes, it is difficult to discern the cause of the departures from the original analyses, but in the case that one or both differences are influencing the current results, it is still clear that the IBS-QOL performs well for IBS-d patients.

The item reduction criteria applied to the 34-item version of the IBS-QOL resulted in many items having high bivariate correlations, as defined as r ≥ 0.7. A possible factor influencing the high correlations between items could be due to priming or order effects, i.e., responses on subsequent items being influenced by earlier-answered items. As the IBS-QOL is a static instrument with only one item order presented to patients, however, testing whether priming influences responses by patients to single items is not possible.

Alternatively, high correlations between items could suggest that the items are measuring a single latent trait. Items 6 (“I feel like I’m losing control of my life because of my bowel problems”), 7 (“I feel my life is less enjoyable because of my bowel problems”), 9 (“I feel depressed about my bowel problems”), and 10 (“I feel isolated from others because of my bowel problems”) all showed a fairly high level of correlation with one another. The α-value for the overall sum scale of the IBS-QOL is also very high, suggesting redundancies across these items.

Similarly, Items 12 (“Because of my bowel problems, sexual activity is difficult for me”) and 20 (“My bowel problems reduce my sexual desire”) exhibited a high correlation with one another (r = 0.741) as expected. Both items make up the Sexual subscale and while the language of the two items respectively imply physical and psychological aspects of sexual activity, patient responses tended to suggest that one does not occur without the other.

There were several other pairs of items that exhibited high inter-item correlation values (cf., Table 1). Our results suggest that a possible future research path for the IBS-QOL is to explore whether a shortened version of the IBS-QOL targeted toward IBS-d could be constructed from the current items while maintaining its measurement properties and still being relevant to IBS-d patients. If items have redundancy, then one could conceive of an item pool that supplies items to each of slightly different versions of the IBS-QOL. Alternatively, specific cognitive debriefing may also help isolate whether any of these items are truly redundant or if items all closely measure HRQOL in IBS-d and simply represent very closely related aspects of IBS-d-related QOL.

Conversely, in IBS-d patients, a departure from the original validation analyses was not surprising either. For example, 72.8% IBS-d patients answered “Not at all” to Item 32 (“I fear I won’t be able to have a bowel movement”) at Baseline. This result fits, conceptually, with how patients should answer items that are not geared toward their IBS subset. This item, therefore, could be taken out of a targeted IBS-d instrument or, perhaps, could simply be included with a binary, “yes” versus “no”, response instead of the 5-point graded response set.

While some of the results suggested that certain items in the IBS-QOL may be candidates to remove if a reduced-item version were to be sought for IBS-d patients, other results support that the full set of items is relevant and psychometrically sound, consistent with conclusions of previous validation studies of the IBS-QOL. This result is not surprising given the extremely high value of Cronbach’s Coefficient α (α = 0.963). This is consistent with the interpretation of the bi-factor and EFA models because the common interpretation of Coefficient α analyses is that the items are internally consistent and therefore represent a unidimensional latent construct.

This conclusion is reinforced by a high observed average item-to-total correlation of 0.642. However, one of the limitations here is that modern applications of α analysis stretch interpretation of the statistic beyond its original intent [28]. Coefficient α was intended to substitute alternate forms reliability—in which two equivalent forms of the instrument were to be administered and the results correlated with one another. As most instrument developers do not have the resources to develop two instruments together, Coefficient α was devised as a means of assessing agreement between an instrument and a theoretical one of same length, comprised of items randomly drawn from all possible content valid items. The coefficient, therefore, is laden with assumptions and also is, ostensibly, a lower bound for the theoretical true internal consistency of a measure. Many have criticized the use of α for this and other reasons [29-31]. Further, while an α assessment assumes sum of item responses, the IBS-QOL standardizes responses to a 0-100 scale, so without further study, it is not clear how the scoring algorithm relates back to a simple sum score. Structural equation modeling techniques, e.g., extensions of the CFA models, actually offer the best alternatives to α and other individual indices as they are better equipped to handle multivariate item data [32,33]. However, any positive or negative bias around the α-value of 0.96 would likely still yield acceptable levels of consistency.

In terms of how the items structurally relate to one another at the instrument level, the fact that the oblique bi-factor model fits the data the best, and better than the orthogonal bi-factor model, suggests that the original factor structure is redundant to the total sum score because factors that are allowed to be correlated fit better with the data than hypothetically independent subscales. We do note, however, that more complex CFA models tended to fit better by both standard fit indexes and usual assessment of residuals and that, generally, increasing model complexity provides better fit in most statistical models. While the oblique bi-factor model accounted for a marginal amount of variance (GFI = 0.8616), an acceptable improvement in variance above a null model (CFI = 0.9073) was observed. The RMSEA index imposes a penalty for higher complexity models, thereby allowing us to infer whether the bi-factor models fit better according to other indices based on their complexity. The observed RMSEA value of 0.069, although moderate in size, comparatively supports the oblique bi-factor conceptualization of IBS-d, i.e., that the best of all CFA models fit is on with an overall latent factor supported by the original substructure whilst allowing the substructure factors to correlate with one another. The model fit may have room for improvement as 10.5% of residuals are outside of the preferred limits potentially indicating that some items may not fit well within the proposed structure.

The EFA model supports that there may be pairs or subsets of items of the IBS-QOL that group together more so than with others—an observation that is not surprising given the observed inter-correlations between items. Interestingly, though, the EFA fit did not produce a factor structure in line with the original substructure, suggesting that HRQOL may be qualitatively different for IBS-d as compared to non-subtyped IBS patients as a whole.

Despite the extraction of multiple factors from the analysis, the EFA fit actually further strengthens the interpretation that the IBS-QOL is unidimensional in IBS-d patients. This is because the EFA model has a large first eigenvalue (37.8) as compared to the second (3.8). Eigenvalues of extracted factors measure the amount of variance observed in the items making up that factor. Here the first extracted factor accounts for 79.5% of the total variance in the items. Further, a test of whether the items suggest a structure in which there is at least one common factor to all items was also significant [χ^2^(561) = 16,080.2, p <0.0001] implying that any structure extracted after that first factor is residual information that could enhance interpretation of the first factor, but one factor would be adequate to interpret the construct under study. This observation indicates that imposing the original factor structure [6] is helping model fit, implying that the original subscale structure of the IBS-QOL seems to be beneficial in accounting for information above and beyond the total sum score. Furthermore, combined with the fit of the orthogonal bi-factor model results, one could conclude for IBS-d patients that the IBS-QOL may be measuring a unidimensional construct, both because of the need to allow factors to correlate and that the original substructure seems only approximately correct.

The CFA and EFA modeling, taken together, suggest that perhaps the best means of assessing the psychometric properties of the IBS-QOL would be to employ Item Response Theory (IRT) methods [34]. IRT approaches estimate a latent construct via a joint model of the individual items. IRT models can also help determine if individual items are performing as intended within the IBS-QOL because relationships between items and the latent trait under study are estimated, directly.

In terms of test-retest reliability, the current analyses demonstrated good levels for the IBS-QOL total score in this regard. Both R_Λ_ and R_T_ exceed the traditionally-accepted reliability threshold of around 0.7 and were comparable to the ICC calculated by the original validation study. Both reliability measures employed here are similar to ICCs with slightly different interpretations. R_Λ_ is the multivariate reliability of the sequence of scores while R_T_ is the average reliability for the total score over any arbitrary number of administrations. Both will tend to increase for a consistent instrument with more administrations because additional information is being taken into account with each added administration. Therefore, with 3 post-Baseline administrations of the IBS-QOL, we have substantial evidence for good reliability of the total score. Contrastingly, even with less information, e.g., two administrations of the IBS-QOL, we would expect that a reliability level would still be approximately 0.75 by our estimates.

The analysis of IBS-QOL total scores with regard to responsiveness were consistent across effect size definitions for different paired comparisons, with moderate increases in effect sizes seen for higher doses of eluxadoline versus placebo. Interestingly, the pattern of effect size estimates suggest that the 100 mg dose of eluxadoline had the largest impact, the same conclusion as was reached by the analysis of clinical measures [5] as defined in FDA’s 2012 IBS Guidance [21]. This conclusion is bolstered by evaluating the cumulative proportions of change from Baseline to Week 12 scores for the IBS-QOL total score with better improvements seen at higher dose levels, specifically 100 and 200 mg. We especially note that within a wide range of improvement levels, the proportion of patients in the eluxadoline 100 mg group meeting given improvements was dramatically higher than those patients receiving placebo. This indicates that the observed treatment effect in the IBS-QOL total score is consistent. Visually, this result is apparent by the wide gap between the placebo and 100 mg eluxadoline lines on Figure 1.

Of note, all treatment groups showed large increases in IBS-QOL total scores at Week 12 as compared to Baseline. Even the Placebo group showed an approximately 17-point increase in total score—higher than the 14-point clinically-significant difference found by Drossman, et al [10]. While further longitudinal study is warranted, we believe that the improvement may be due to natural cycling of disease or due to potential Hawthorne effects, ie, improvements by patients as a result of simply being observed. We do, however, also note that the treatment group differences approximate a dose response that peaks at 100 mg and plateaus with 200 mg. This pattern mimics that of the other outcome measures reported elsewhere [5].

Our analyses suggest that a reduced-form IBS-QOL specific for IBS-d sufferers may improve measurement of IBS-related QOL for these patients. However, further research is necessary to determine which of the items may be ideally suited for a reduced form. We suggest that a better characterization of item-level properties of the IBS-QOL via IRT methodology would be helpful in determining an optimal item configuration.

## Conclusions

Much of the original development and validation work on the IBS-QOL items were replicated in the current paper. However, some items do not appear perform ideally for IBS-d patients, either individually or with one another, and a reduced-item set for the IBS-QOL may produce better overall measurement of the IBS-d condition. Despite some indications of improvements that could be made, the current 34-item instrument does work in IBS-d patients; it performs well on the classical set of psychometric assessments and is demonstrated to be: Approximately unidimensional as evidenced by the high first eigenvalue extracted from the EFA model as well as the high Coefficient α value observed among the items; reliable as evidenced by the consistently high R_Λ_ and R_T_ values; and correlated with other measures of IBS-d symptoms and outcomes—both new, like the FDA Clinical Responders, and old, i.e., IBS-AR. Combined with good ability to detect change, evidenced by moderately high effect sizes in changes from Baseline to Week 12 and good discrimination of the 100 mg dose versus placebo over a wide range of improvement levels—cf. Figure 1—we believe that the IBS-QOL total score is a psychometrically valid means of assessing QOL in IBS-d patients.

There are indications that the individual items do contain more information than what is expressed in a sum or scaled total score. Thus, directly relating items to the latent construct of IBS-d-specific QOL via IRT modeling should be considered for future research on the IBS-QOL to determine if there are untapped measurement properties within the items. Taken together, the current results suggest the IBS-QOL is a psychometrically sound instrument for patients with diarrhea predominant IBS and the total score is a good, unified measure of HRQOL. Importantly, all of the results together suggest that the IBS-QOL appears to detect the core concepts of IBS-d as well as changes in the disease state. Further, the relatively high observed correlations between the IBS-QOL and other established efficacy measures reinforces the conclusion that the IBS-QOL is not only a reliable, but also a valid and sensitive measure of patients’ IBS-d experiences.

## Abbreviations

AIC: Akaike Information Criterion; BIC: Bayesian Information Criterion; CFA: Confirmatory factor analysis; CFI: Comparative Fit Index; DF: Degrees of freedom; δ-OR: Delta opioid receptor; EFA: Exploratory factor analysis; FDA: Food and Drug Administration; GFI: Goodness of Fit Index; HRQOL: Health-related quality of life; ICC: Intraclass correlation; IBS: Irritable bowel syndrome; IBS-AR: Irritable Bowel Syndrome Adequate Relief; IBS-c: Irritable bowel syndrome—constipation subtype; IBS-d: Irritable bowel syndrome—diarrheal subtype; IBS-m: Irritable bowel syndrome—mixed subtype; IBS-QOL: Irritable Bowel Syndrome Quality of Life questionnaire; IBS-SSS: Irritable Bowel Syndrome Symptom Severity Score; IRT: Item response theory; μ-OR: Mu opioid receptor; PCA: Principal components analysis; RMSEA: Root mean squared error of approximation; WAP: Worst abdominal pain.

## Competing interests

The current project was funded by Furiex Pharmaceuticals. DAA and PSC are employees of Furiex and both own stock in the company. DLP and DAD are paid consultants to Furiex.

## Authors’ contributions

DAA contributed to the trial design, conceived the analysis strategy, analyzed the data, and drafted the manuscript. DLP and DAD aided in interpretation of the data and provided critical input to the manuscript development. PSC helped design the clinical trial from which the data were generated, edited and gave significant commentary to the content, and aided in drafting the manuscript. All authors read and approved the final manuscript.

## Supplementary Material

Additional file 1 The IBS-QOL Questionnaire. Click here for file

Additional file 2 Study Investigators. Click here for file

Additional file 3: Figure S1 Structure Diagrams for the IBS-QOL Total Score Factor Models. Click here for file

Additional file 4 Assessing Reliability by Estimating Variances using Linear Models. Click here for file

Additional file 5 Biserial Correlations. Click here for file

Additional file 6: Table S1 Item-total Correlations and Overall Coefficient α. Click here for file
