# Supplementary material for: Evaluation of the Irritable Bowel Syndrome Quality of Life (IBS-QOL) questionnaire in diarrheal-predominant irritable bowel syndrome patients
Source: Health Qual Life Outcomes. 2013 Dec 13;11:208. doi: 10.1186/1477-7525-11-208 (PMC3895767; doi:10.1186/1477-7525-11-208)
Supplement: Additional file 1 — The IBS-QOL Questionnaire. [file 1477-7525-11-208-S1.pdf]

# Supplemental Appendix A: The IBS-QOL Questionnaire

About how you feel

Please think about your life over the past month (last 30 days), and look at the statements below. Each statement has five different responses. For each statement, please circle the response that best describes your feelings.

Q1. I feel helpless because of my bowel problems.
(Please circle one number)
1 NOT AT ALL
2 SLIGHTLY
3 MODERATELY
4 QUITE A BIT
5 EXTREMELY

Q2. I am embarrassed by the smell caused by my bowel problems.
(Please circle one number)
1 NOT AT ALL
2 SLIGHTLY
3 MODERATELY
4 QUITE A BIT
5 EXTREMELY

Q3. I am bothered by how much time I spend on the toilet.
(Please circle one number)
1 NOT AT ALL
2 SLIGHTLY
3 MODERATELY
4 QUITE A BIT
5 A GREAT DEAL

Q4. I feel vulnerable to other illnesses because of my bowel problems.
(Please circle one number)
1 NOT AT ALL
2 SLIGHTLY
3 MODERATELY
4 QUITE A BIT
5 EXTREMELY

Q5. I feel fat/bloated because of my bowel problems.
(Please circle one number)
1 NOT AT ALL
2 SLIGHTLY
3 MODERATELY
4 QUITE A BIT
5 A GREAT DEAL

Q6. I feel like I'm losing control of my life because of my bowel problems.
(Please circle one number)
1 NOT AT ALL
2 SLIGHTLY
3 MODERATELY
4 QUITE A BIT
5 A GREAT DEAL

Q7. I feel my life is less enjoyable because of my bowel problems.
(Please circle one number)
1 NOT AT ALL
2 SLIGHTLY
3 MODERATELY
4 QUITE A BIT
5 A GREAT DEAL

Q8. I feel uncomfortable when I talk about my bowel problems.
(Please circle one number)
1 NOT AT ALL
2 SLIGHTLY
3 MODERATELY
4 QUITE A BIT
5 EXTREMELY

Q9. I feel depressed about my bowel problems.
(Please circle one number)
1 NOT AT ALL
2 SLIGHTLY
3 MODERATELY
4 QUITE A BIT
5 EXTREMELY

Q10. I feel isolated from others because of my bowel problems.
(Please circle one number)
1 NOT AT ALL
2 SLIGHTLY
3 MODERATELY
4 QUITE A BIT
5 EXTREMELY

Q11. I have to watch the amount of food I eat because of my bowel problems.
(Please circle one
number)
1 NOT AT ALL
2 SLIGHTLY
3 MODERATELY
4 QUITE A BIT
5 A GREAT DEAL

Q12. Because of my bowel problems, sexual activity is difficult for me.
(Please circle one number)
(If not applicable, please circle “NOT AT ALL”)
1 NOT AT ALL
2 SLIGHTLY
3 MODERATELY
4 QUITE A BIT
5 EXTREMELY

Q13. I feel angry that I have bowel problems.
(Please circle one number)
1 NOT AT ALL
2 SLIGHTLY
3 MODERATELY
4 QUITE A BIT
5 EXTREMELY

Q14. I feel like I irritate others because of my bowel problems.
(Please circle one number)
1 NOT AT ALL
2 SLIGHTLY
3 MODERATELY
4 QUITE A BIT
5 A GREAT DEAL

Q15. I worry that my bowel problems will get worse.
(Please circle one number)
1 NOT AT ALL
2 SLIGHTLY
3 MODERATELY
4 QUITE A BIT
5 A GREAT DEAL

Q16. I feel irritable because of my bowel problems.
(Please circle one number)
1 NOT AT ALL
2 SLIGHTLY
3 MODERATELY
4 QUITE A BIT
5 EXTREMELY

Q17. I worry that people think I exaggerate my bowel problems.
(Please circle one number)
1 NOT AT ALL
2 SLIGHTLY
3 MODERATELY
4 QUITE A BIT
5 A GREAT DEAL

Q18. I feel I get less done because of my bowel problems.
(Please circle one number)
1 NOT AT ALL
2 SLIGHTLY
3 MODERATELY
4 QUITE A BIT
5 A GREAT DEAL

Q19. I have to avoid stressful situations because of my bowel problems.
(Please circle one number)
1 NOT AT ALL
2 SLIGHTLY
3 MODERATELY
4 QUITE A BIT
5 A GREAT DEAL

Q20. My bowel problems reduce my sexual desire.
(Please circle one number)
1 NOT AT ALL
2 SLIGHTLY
3 MODERATELY
4 QUITE A BIT
5 A GREAT DEAL

Q21. My bowel problems limit what I can wear.
(Please circle one number)
1 NOT AT ALL
2 SLIGHTLY
3 MODERATELY
4 QUITE A BIT
5 A GREAT DEAL

Q22. I have to avoid strenuous activity because of my bowel problems.
(Please circle one number)
1 NOT AT ALL
2 SLIGHTLY
3 MODERATELY
4 QUITE A BIT
5 A GREAT DEAL

Q23. I have to watch the kind of food I eat because of my bowel problems.
(Please circle one number)
1 NOT AT ALL
2 SLIGHTLY
3 MODERATELY
4 QUITE A BIT
5 A GREAT DEAL

Q24. Because of my bowel problems, I have difficulty being around people I do not know well.
(Please circle one number)
1 NOT AT ALL
2 SLIGHTLY
3 MODERATELY
4 QUITE A BIT
5 A GREAT DEAL

Q25. I feel sluggish because of my bowel problems.
(Please circle one number)
1 NOT AT ALL
2 SLIGHTLY
3 MODERATELY
4 QUITE A BIT
5 EXTREMELY

Q26. I feel unclean because of my bowel problems.
(Please circle one number)
1 NOT AT ALL
2 SLIGHTLY
3 MODERATELY
4 QUITE A BIT
5 EXTREMELY

Q27. Long trips are difficult for me because of my bowel problems.
(Please circle one number)
1 NOT AT ALL
2 SLIGHTLY
3 MODERATELY
4 QUITE A BIT
5 EXTREMELY

Q28. I feel frustrated that I cannot eat when I want because of my bowel problems.
(Please circle one number)
1 NOT AT ALL
2 SLIGHTLY
3 MODERATELY
4 QUITE A BIT
5 EXTREMELY

Q29. It is important to be near a toilet because of my bowel problems.
(Please circle one number)
1 NOT AT ALL
2 SLIGHTLY
3 MODERATELY
4 QUITE A BIT
5 EXTREMELY

Q30. My life revolves around my bowel problems.
(Please circle one number)
1 NOT AT ALL
2 SLIGHTLY
3 MODERATELY
4 QUITE A BIT
5 A GREAT DEAL

Q31. I worry about losing control of my bowels.
(Please circle one number)
1 NOT AT ALL
2 SLIGHTLY
3 MODERATELY
4 QUITE A BIT
5 A GREAT DEAL

Q32. I fear that I won't be able to have a bowel movement.
(Please circle one number)
1 NOT AT ALL
2 SLIGHTLY
3 MODERATELY
4 QUITE A BIT
5 A GREAT DEAL

Q33. My bowel problems are affecting my closest relationships.
(Please circle one number)
1 NOT AT ALL
2 SLIGHTLY
3 MODERATELY
4 QUITE A BIT
5 A GREAT DEAL

Q34. I feel that no one understands my bowel problems.
(Please circle one number)
1 NOT AT ALL
2 SLIGHTLY
3 MODERATELY
4 QUITE A BIT
5 EXTREMELY
