# Supplementary material for: Evaluation of the Irritable Bowel Syndrome Quality of Life (IBS-QOL) questionnaire in diarrheal-predominant irritable bowel syndrome patients
Source: Health Qual Life Outcomes. 2013 Dec 13;11:208. doi: 10.1186/1477-7525-11-208 (PMC3895767; doi:10.1186/1477-7525-11-208)
Supplement: Additional file 2 — Study Investigators. [file 1477-7525-11-208-S2.pdf]

Study Investigators

Adel N. Abdelsayed, Aaron Adaoag, Dilawar Ajani, Robert P. Albares, Teresa Alfonso, Mohammed A. Allaw, Stephen T. Amann, Charles P. Andrews, Royal B. Anspach, Avni Arora, Armen Arslanian, John T. Baber, Ian M. Baird, Jeffrey B. Baker, Anezi E. Bakken, Natarajan Bala, Krishnasomy Balakrishnan, Leslie Bank, Mark A. Barber, Robert J. Bargar, Charles F. Barish, James Barker, Mira Baron, Richard L. Beasley, Jay G. Beckwith, Gary Bedel, Simon Behar, Jeffrey S. Ben Zvi, Jennifer Berry, Roger D. Beyer, Raj Bhandari, Barry M. Blumenthal, Verle D. Bohman, Donald M. Brandon, Donald P. Brannan, Robert W. Braun, Elizabeth Bretton, Curtis L. Brown, Dennis K. Buth, Charles Cattano, Louis B. Chaykin, Randall Chee-Awai, Deanna G. Cheung, Steve H. Choi, Shane Christensen, Neil M. Cohen, Barry G. Collins, Lisa B. Connery, Paula Lane, Yvette G. Crabtree, Israel Crespo, Adnan Dahdul, Amador S. Delamerced, Paul A. Deneault, Donna M. DeSantis, Michael C. DeSantis, Robert Detweiler, Meera A. Dewan, Dinh V. Dinh, Richard Dobrusin, Michael T. Draelos, Douglas A. Drossman, Steven Duckor, David A. Dulitz, Michael J. Dunn, Ervin Y. Eaker, William T. Ellison, Atilla Ertan, James T. Farrell, Ramin H. Farsad, Steven A. Fein, Agustin Fernandez, R. David Ferrera, Thomas C. Fiel, Matthew Finneran, Gregory M. Flippo, Ronald P. Fogel, Steven A. Foley, Fred Fowler, Keith A. Friedenberg, Joseph Healy, John D. Gabriel, Syam P. Gaddam, Barry M. Garner, Michael M. Gaspari, Prodyot Ghosh, Lev Ginsburg, Carl A. Goetsch, Michael Goldstein, Jeff Gonzalez, Glenn L. Gordon, David Grant, Craig Gross, Michael R. Grossman, Alfredo Gueler, Michael C. Hagan, Richard S. Hamilton, Robert A. Han, Robert Hardi , Paul A. Hellstern, Dan C. Henry, Robert J. Holmes, Douglas Homoky, C. S. Horn, Kevin B. Horton, Cynthia L. Huffman, Sergio Ibarra, Ikechukwu E. Ibegbu, Aamir Z. Jamal, Francis Jimenez, William L. Jonakin, Katie A. Julien, Rashid A. Khairi, Jeffrey J. Kaladas, Marvin Kalafer, Karen E. Karn, David B. Kaner, Robert Kaplan, Pankaj K. Kashyap, Shaun B. Kelehan, Susan E. Kemp, John W. Kessel, Sardar Khan, Muhammad A. Khan, Andrew C. Kim, Murray A. Kimmel, Judith L. Kirstein, Stanley Koch, Valli Kodali, Daniel Kohm, James H. Kopp, Richard A. Krause, Pradeep Kumar, Mark E. Kutner, Gigi C. Lefebvre, Anthony Lembo, Kurt W. Lesh, Scott Levenson, Mark A. Lijewski, Michael J. Lillestol, Joseph L. Lillo, Donald C. Lipkis, Thomas A. Loiudice, Daniel G. Lorch, John Lowe, Pramod Malik, Nasrullah Manji, Stefano Marcuard, Maria C. Mascola, Brock A. McConnehey, Diane O. McConnehey, Richard McDavid, Carl R. Meisner, James M. Mertsdorf, Sharon L. Miller, Philip B. Miner, Jr., Stephen M. Minton, Matthew J. Mlot, Nancy J. Morgan, Mark Nagrani, Fadel E. Nammour, Shardul Nanavati, Shahila Navayogarajah, Aris Nikas, Thomas V. Nowak, Thomas O'Barr, Mandeep S. Oberoi, David Oelsner, Adebambo Ojuri, Joanne G. Pagal, Henry Paik, John E. Pappas, Dipti Patel, Pravinchandra H. Patel, Lisa Pathak, Walter S. Patton, Leonel Perez-Limonte, Lenin J. Peters, Carlos Petit, Michelle A. Petro, Michael A. Pezzone, Raymond W. Phillips, Humberto Aguilar, Bryan Pogue, Adrienne L. Prentiss, Mitchell A. Pressman, Clifton J. Prince, Charles W. Randall, Bruce G. Rankin, Babu V. Rao,

Donald Rauh, Roderick A. Remoroza, Harvey Resnick, Hubert Reyes, Donato R. Ricci, Dennis Riff , Ernie Riffer, Mark A. Ringold, Robert Ringrose, Timothy Ritter, Michael Robinson, John Rubino, Frederick W. Ruthardt, Shahriar S. Safavi, Nisal K. Samarasekera, Robert J. Saniuk, Jose A. Santiago, William O. Sargeant, Leah M. Schmidt, Jeffrey H. Schneider, Michael E. Schwartz, Shahriar Sedghi, Barry Seidman, Thomas E. Sepe, Harry Serfer, Umedchandra K. Shah, Atul R. Shah, Smita S. Shah, Larry J. Sharp, Alex Sherman, Ann L. Silverman, Timothy C. Simmons, Russell W. Simpson, Steve Sitar, Teresa S. Sligh, Timothy R. Smith, David Smith, Alexander R. Smythe II, Joseph Soufer, Jack Spainhour, John Speer, Joseph F. Staffetti, Mary L. Stedman, Lawrence Stein, Richard B. Stewart, Neil Stollman, Cynthia B. Strout, Robert A. Strzinek, Elhan Suley, Sigrid Sanchez, Farid Taie, Gregory P. Tarleton, Samuel O. Teniola, Orlando F. Torres, Douglas M. Trate, Andrzej Triebling, Mark Turner, Kari T. Uusinarkaus, Erik Van Ginkel, Rajeev Vasudeva, Ravikumar Vemuru, Alan Wanderer, Elise R. Wiesner, Hayes Williams, Scott A. Wilson, Barry Winston, John Wo, Lawrence Wruble, David J. Wyatt, Robert Yin, Ziad Younes, Salam Zakko, Andrew Zwick
