# Supplementary material for: Evaluation of the Irritable Bowel Syndrome Quality of Life (IBS-QOL) questionnaire in diarrheal-predominant irritable bowel syndrome patients
Source: Health Qual Life Outcomes. 2013 Dec 13;11:208. doi: 10.1186/1477-7525-11-208 (PMC3895767; doi:10.1186/1477-7525-11-208)

Supplemental Figure 1. Structure Diagrams for the IBS-QOL Total Score Factor Models

A. Original IBS-QOL Substructure

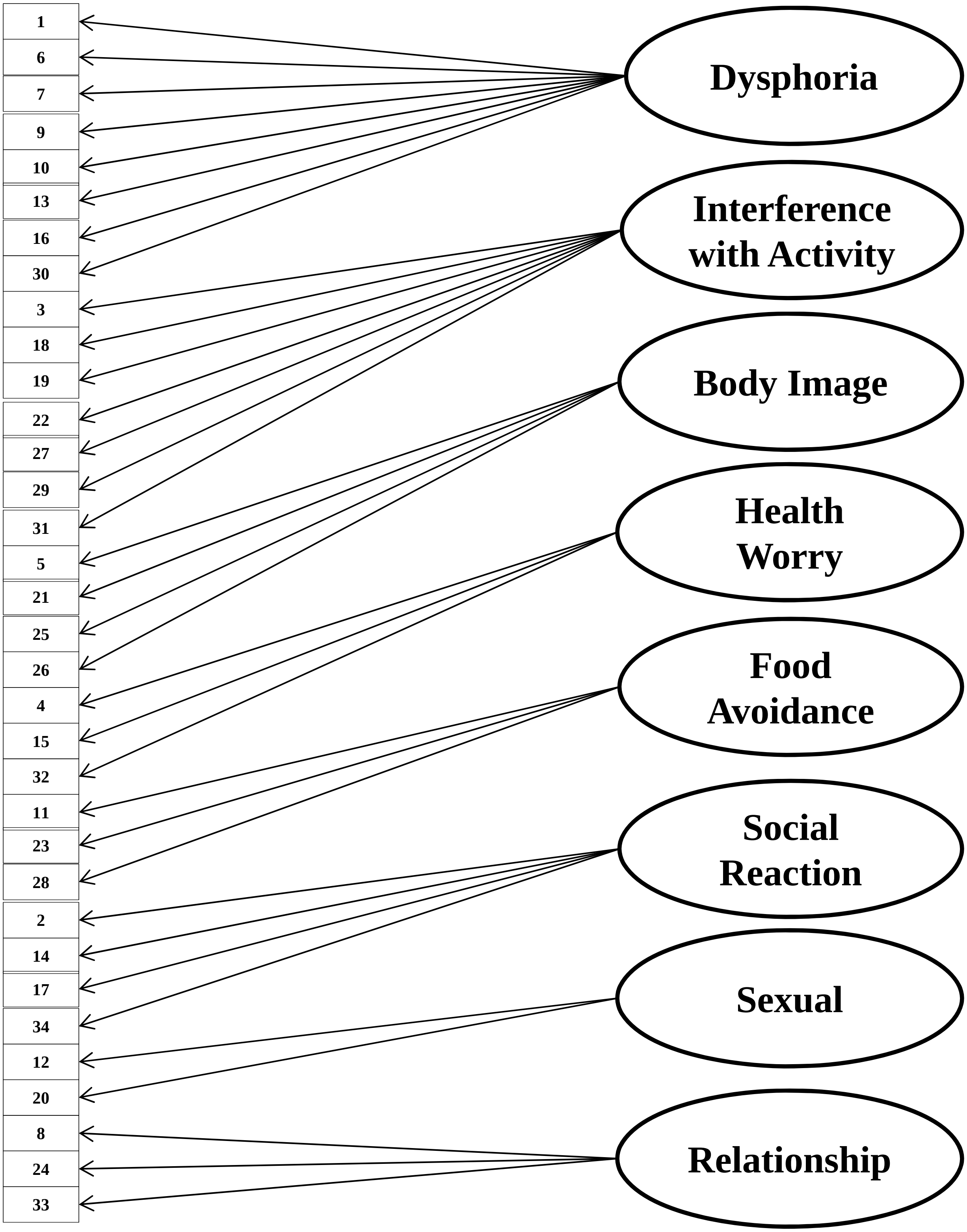

B. Hierarchical IBS-QOL Substructure

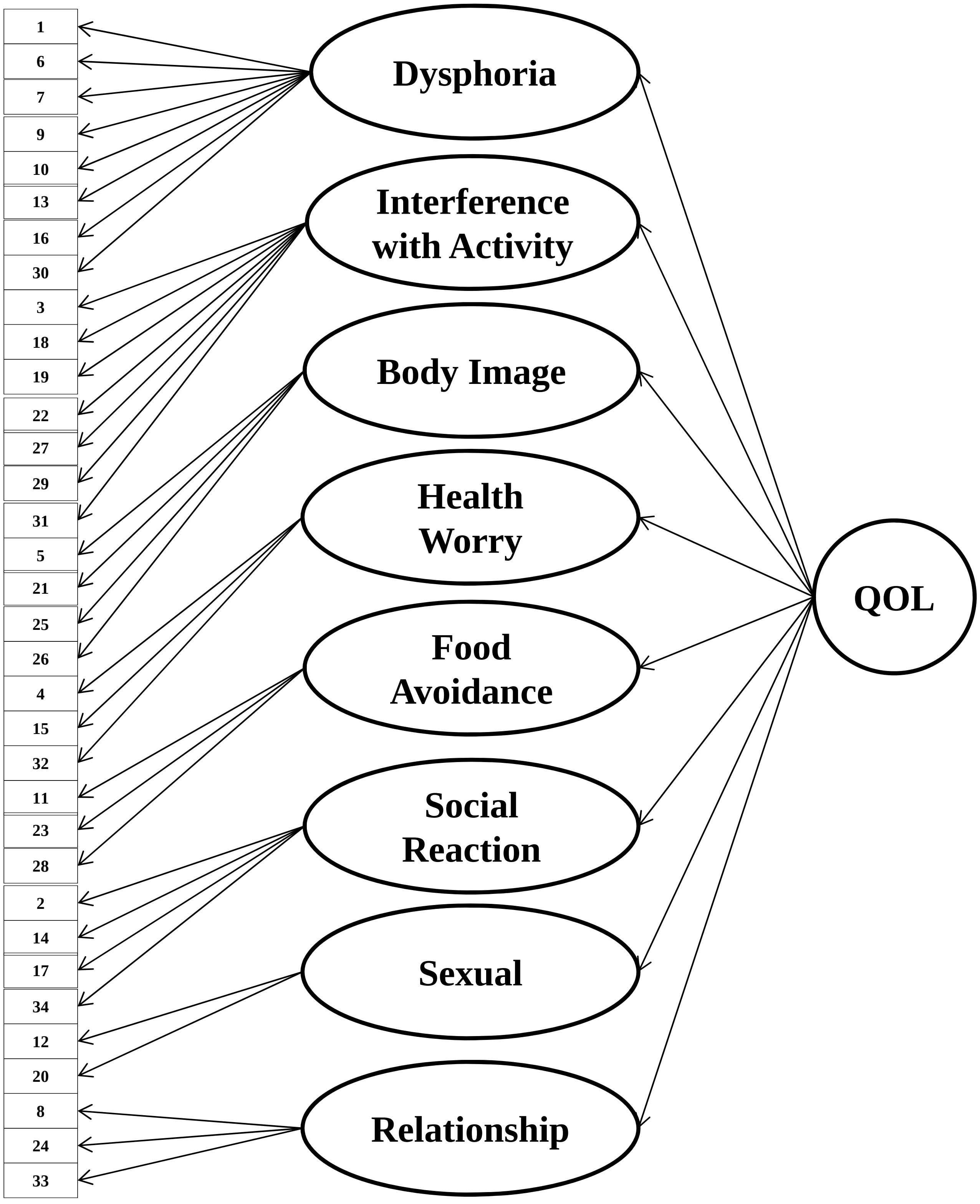

C. Bifactor Structure

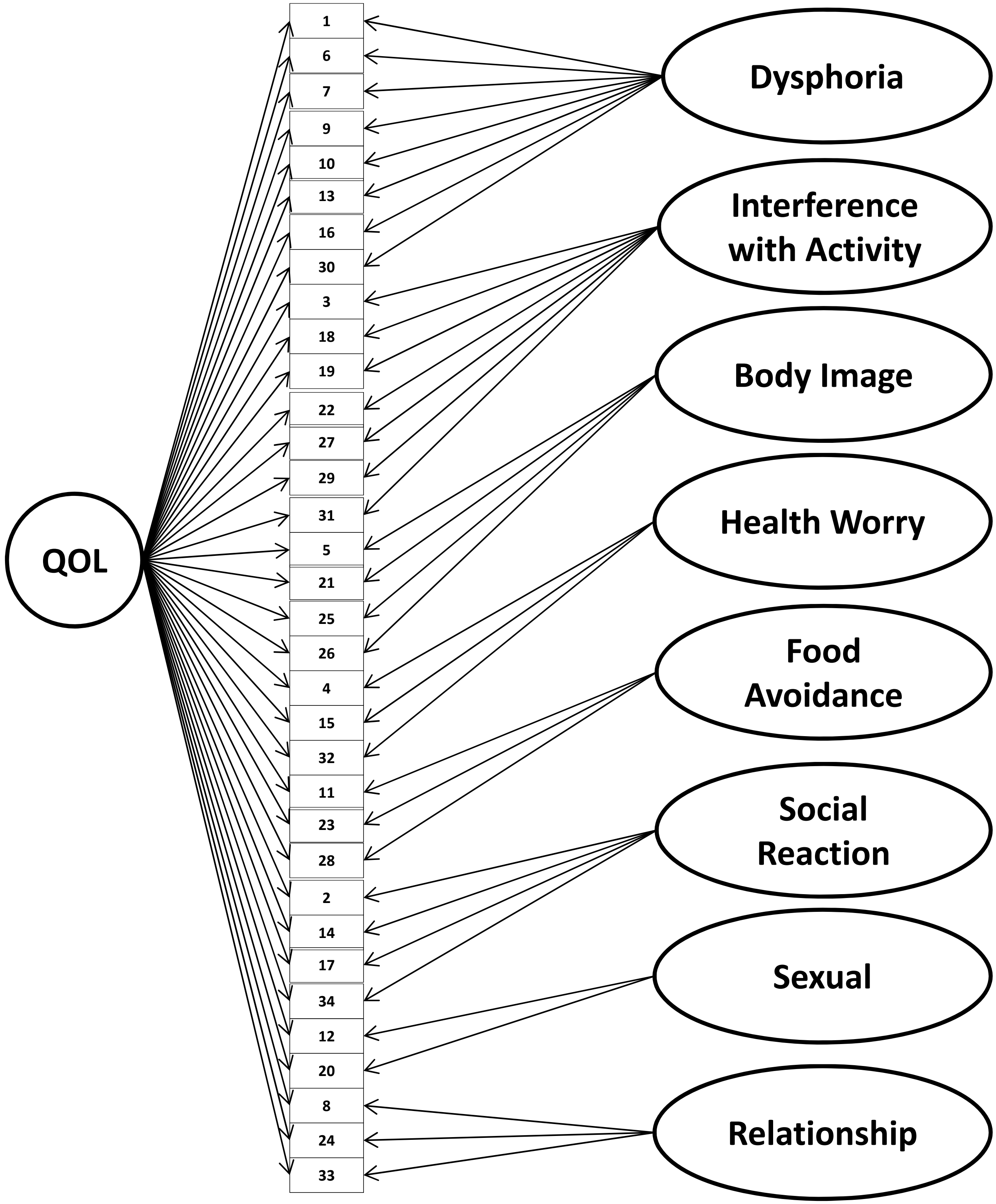

D. Single Factor Structure

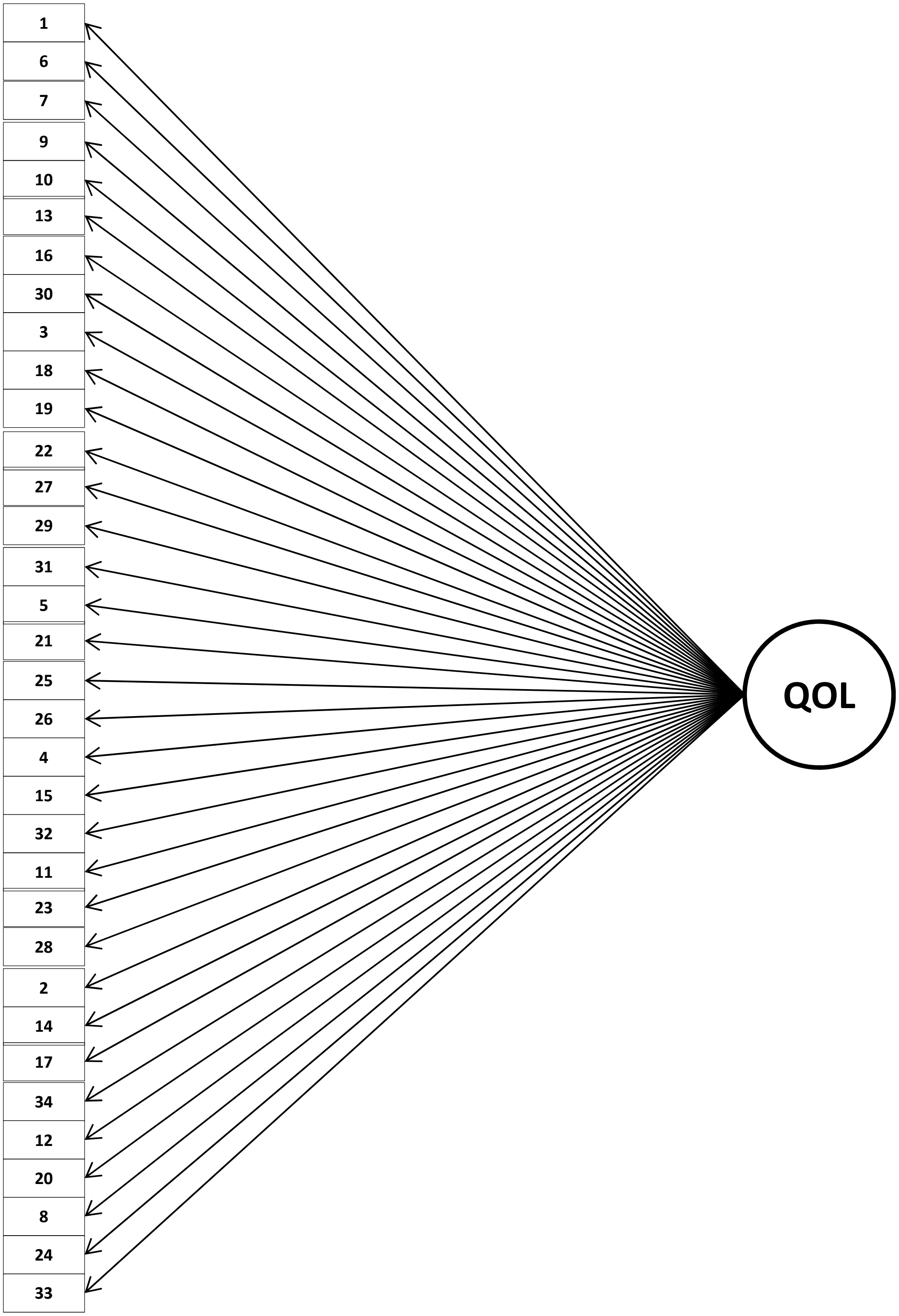

Supplement: Additional file 3: Figure S1 — Structure Diagrams for the IBS-QOL Total Score Factor Models. [file 1477-7525-11-208-S3.pdf]
