# Supplementary material for: Evaluation of the Irritable Bowel Syndrome Quality of Life (IBS-QOL) questionnaire in diarrheal-predominant irritable bowel syndrome patients
Source: Health Qual Life Outcomes. 2013 Dec 13;11:208. doi: 10.1186/1477-7525-11-208 (PMC3895767; doi:10.1186/1477-7525-11-208)
Supplement: Additional file 4 — Assessing Reliability by Estimating Variances using Linear Models. [file 1477-7525-11-208-S4.pdf]

Supplemental Appendix B: Assessing Reliability by Estimating Variances using Linear Models

The variances used to calculate R_Λ are estimated in the longitudinal mixed effects model:

$$Y_i = \mu + X_i\beta + Z_i\gamma + \varepsilon_r$$

where Y_i is the vector of responses for patient i, X_i and Z_i are matrices of the known covariates, β is a matrix of fixed effects, γ ~ Gaussian(0,G) is a matrix of random effects and ε_r ~ Gaussian(0, Σ_r) is a matrix of residual effects that specify the repeated measurement structure. The longitudinal mixed-effects model was fit with main effects for treatment group (5 mg BID, 25 mg BID, 100 mg BID, 200 mg BID, and Placebo), time in months (1, 2, and 3 months post-Baseline), and the Baseline value of the IBS-QOL as fixed effects. The interaction term for treatment and time as well as a quadratic term for time were also fit. Additionally, random effects were fit for the intercept and time and a residual effect for time was further fit to partition variance associated with the correlation between repeated administrations of the IBS-QOL.

The R_Λ statistic[1] was then calculated where

$$R_\Lambda = 1 - \left|\Sigma_R V^{-1}\right|$$

is similar to the Wilks' Λ statistic as well as the classical definition of reliability, albeit in matrix form. In the above equation, Σ_R is a p × p matrix for p time points and it represents the residual variability, i.e., error, due to effects of the longitudinal model; V is a p × p matrix of elements that represent the total observed variance. The matrix $\Sigma_R V^{-1}$, therefore, contains elements that represent the ratio of variance not accounted for by the model to the total observed variance in the data. For example, the interpretation of the $\Sigma_R V^{-1}$ matrix is that it contains available information about the measurement of the latent constructs within the data. By estimating the ΣV^{-1} and other matrices via the mixed effects model above, variables that could affect traditional ICC measurements, e.g., different treatments, can be accounted for, reducing any inherent bias in the estimation of reliability.

The determinant of ΣV^{-1}, then, is an estimate of the variability within the p × p elements that comprise the matrix.Therefore, summarization of the matrix provides the basis for test statistics that allow inferences about the consistency of the instrument, e.g., the IBS-QOL, over repeated measurements, but since the linear model accounts for treatment and time effects the R_Λ statistic is controlled for these effects over the repeated measurements of the IBS-QOL. R_Λ can be considered as the joint reliability over the course of repeated measurements representing the concept that as more information is collected, more is known about the instrument—a concept that is inherent to Coefficient α as well.

The similar measure, R_T[2] was also calculated:

$$R_T = 1 - \frac{\mathrm{trace}(\Sigma_R)}{\mathrm{trace}(V)}$$

where Σ_R and V are defined similarly to above. R_T is similar to R_Λ, however, it represents a quantity akin to the average reliability over repeated measurements of an instrument, conditional on the longitudinal model fit. Both R_Λ and R_T were computed on the IBS-QOL total score to assess reproducibility of scores over repeated administrations.

References

1. Laenen A, Alonso A, Molenberghs G, Vangeneugden T. **Reliability of a Longitudinal Sequence of Scale Ratings.** *Psychometrika*. 2009;**74**:49-64.

2. Laenen A, Alonso A, Molenberghs G. A **Measure for the Reliability of a Rating Scale Based on Longitudinal Clinical Trial Data.** *Psychometrika*. 2007;**72**:443-448.
