# Supplementary material for: Evaluation of the Irritable Bowel Syndrome Quality of Life (IBS-QOL) questionnaire in diarrheal-predominant irritable bowel syndrome patients
Source: Health Qual Life Outcomes. 2013 Dec 13;11:208. doi: 10.1186/1477-7525-11-208 (PMC3895767; doi:10.1186/1477-7525-11-208)
Supplement: Additional file 5 — Biserial Correlations. [file 1477-7525-11-208-S5.pdf]

Supplemental Appendix C: Biserial Correlations

The point-biserial correlation[1] is defined by the following equation

$$r_{pbs} = \frac{\mu_{(+)} - \mu_{(-)}}{s_{Tx}} \sqrt{\pi_{(+)}\pi_{(-)}}$$

where μ_i are the IBS-QOL Total Score mean values for those who report adequate relief or no relief at Week 12, respectively, s_{Tx} is the standard deviation accounting for dose level, and π_i are the proportions of patients who reported adequate relief or no relief, respectively. This calculation was used to assess the linear relationship between IBS-QOL total score and the IBS-AR. The resultant correlation estimate assumes a natural dichotomy and assesses the linear relationship, in the current case, between the IBS-AR probability of relief or FDA Clinical Responder status and means of the IBS-QOL total score at Week 12. The formula above also accounts for treatment dose by using the standard deviation of the IBS-QOL at Week 12, accounting for dose level received.

The biserial correlation[2] was also calculated and is given by the formula

$$r_{bis} = \frac{\mu_{(+)} - \mu_{(-)}}{s_{Tx} Z_p}$$

with similar definitions as above and

$$Z_p = \frac{1}{\sqrt{2\pi}} \exp\left\{ -\frac{1}{2} \Phi^{-1}\left[\pi_{(-)}\right]^2 \right\}$$

representing the ordinate of the standard Gaussian distribution, $\pi_{(-)}$ indicating the proportion of patients who answered the item denoting "No Relief" for IBS-AR, being a nonresponder for the FDA Clinical Responder definition, and $\Phi^{-1}(\cdot)$ representing the inverse of the Gaussian distribution for the threshold for positive response, i.e., below which patients report a negative response and above which they report a positive response. The biserial correlation assumes that the dichotomous variable follows an underlying Gaussian distribution. To assess statistical significance of the r_{pbs} and r_{bis} statistics, t-tests will be constructed via the following formula

$$t_i = r_i \sqrt{\frac{N}{1 - r_i^2}}$$

evaluated with (N – 2) degrees of freedom and i indexing point biserial versus biserial.

References

1. Das Gupta S: **Point biserial correlation and its generalization**. *Psychometrika*. 1960;**25**:393-408.

2. Brogden H: **A new coefficient: Application to biserial correlation and to estimation of selective efficiency**. *Psychometrika*. 1949;**14**:169-182.
