# Supplementary material for: Evaluation of the Irritable Bowel Syndrome Quality of Life (IBS-QOL) questionnaire in diarrheal-predominant irritable bowel syndrome patients
Source: Health Qual Life Outcomes. 2013 Dec 13;11:208. doi: 10.1186/1477-7525-11-208 (PMC3895767; doi:10.1186/1477-7525-11-208)
Supplement: Additional file 6: Table S1 — Item-total Correlations and Overall Coefficient α. [file 1477-7525-11-208-S6.pdf]

Supplemental Table 1. Item-total Correlations and Overall Coefficient α

Item	Correlation with Total
1. I feel helpless because of my bowel problems.	0.68
2. I am embarrassed by the smell caused by my bowel problems.	0.52
3. I am bothered by how much time I spend on the toile t.	0.63
4. I feel vulnerable to other illnesses because of my bowel problems.	0.63
5. I feel fat because of my bowel problems.	0.47
6. I feel like I'm losing control of my life because of my bowel problems.	0.76
7. I feel my life is less enjoyable because of my bowel problems.	0.72
8. I feel uncomfortable when I talk about my bowel problems.	0.55
9. I feel depressed about my bowel problems.	0.73
10. I feel isolated from others because of my bowel problems.	0.80
11. I have to watch the amount of food I eat because of my bowel problems.	0.56
12. Because of my bowel problems, sexual activity is difficult for me.	0.58
13. I feel angry that I have bowel problems.	0.68
14. I feel like I irritate others because of my bowel problems.	0.70
15. I worry that my bowel problems will get worse.	0.65
16. I feel irritable because of my bowel problems.	0.73
17. I worry that people think I exaggerate my bowel problems.	0.62
18. I feel I get less done because of my bowel problems.	0.75
19. I have to avoid stressful situations because of my bowel problems.	0.64
20. My bowel problems re duce my sexual desire.	0.57
21. My bowel problems limit what I can wear.	0.65
22. I have to avoid strenuous activity because of my bowel problems.	0.65
23. I have to watch the kind of food I eat because of my bowel problems.	0.51
24. Because of my bowel problems, I have difficulty being around people I do not know well.	0.69
25. I feel sluggish because of my bowel problems.	0.67
26. I feel unclean because of my bowel problems.	0.66
27. Long trips are difficult for me because of my bowel problems.	0.67
28. I feel frustrated that I cannot eat when I want because of my bowel problems.	0.68
29. It is important to be near a toile t because of my bowel problems.	0.65
30. My life revolves around my bowel problems.	0.77
31. I worry about losing control of my bowels.	0.68
32. I fear that I won't be able to have a bowel movement.	0.29
33. My bowel problems are affecting my closest relationships.	0.65
34. I feel that no one understands my bowel problems.	0.66
Overall Item-total Correlation (95% CI)	0.64 (0.609, 0.677)
Coefficient α (95% CI)	0.963 (0.959, 0.966)
